# Deaths from Chloral Hydrate

**Published:** 1871-09

**Authors:** 


### Deaths from Chloral Hydrate.

Dr. H. W. Fuller describes a number of cases in which alarming effects followed the exhibition of 30 grains of chloral, besides one fatal one. The latter occurred in a young woman, previously in good health, to whom the remedy was prescribed for hysterical symptoms occurring at the menstrual period. The dose was taken at 10 A.M. She immediately became much excited, and complained of pain in the chest. In an hour she fell asleep, slept heavily all night, and continued so doing all the next morning, although every effort was made by the medical attendant to rouse her. When first seen by the latter respiration was deep and sighing, the pulse imperceptible at the wrist. Under the use of external and internal stimulents the pulse became slightly apparent. At 2 P M., when Dr. Fuller saw her, she was very pale and cold, with widely-dilated, very sluggish pupils, and heavy, distinctly sighing breathing; the pulse was barely perceptible, the heart beating 120 a minute, with clear sounds of normal rhythm, although its action was manifestly very weak. The abdomen was flat and soft, the limbs moderately relaxed. In spite of active stimulation she died the next morning, without ever having moved a muscle or shown the slightest sign of consciousness after having fallen asleep.—*London Lancet, March* 25, 1871.

---

Mr. H. Norris reports the case of a woman, violent, hysterical, dipsomaniac, who took 712 grains in nine days, 260 grains of this being taken in the last 35 hours. In the evening of the day preceding her death she had been out to tea; between 10 and 11 P.M. she took 40 grains, and 1 and 2 A.M. 36 grains, and at day-light another 36 grains. Between 10 and 11 A.M. awoke and went down stairs to breakfast; about 12 was found in bed, vomiting; was left five minutes, and was then found dead on the floor. At the autopsy no smell of chloroform could be detected. The heart was found somewhat pallid; the ventricles empty; the auricles partially distended by dark semi-coagulated blood. The stomach, liver, heart, kidney, and spleen were chemically examined one hundred and thirty hours after death. They were remarkably well preserved. On distillation with caustic soda, chloroform was obtained from the contents of the stomach and from the liver, but not from the tissues —*London Lancet.—New Remedies.*

